# Supplementary material for: Hierarchical Nanocauliflower Chemical Assembly Composed of Copper Oxide and Single-Walled Carbon Nanotubes for Enhanced Photocatalytic Dye Degradation
Source: Nanomaterials (Basel). 2021 Mar 10;11(3):696. doi: 10.3390/nano11030696 (PMC7998431; doi:10.3390/nano11030696)
Supplement: Supplementary file 1 [file nanomaterials-11-00696-s001.pdf]

nanomaterials

Supplementary Materials

Hierarchical Nanocauliflower Chemical Assembly Composed of Copper Oxide and Single-Walled Carbon Nanotubes for Enhanced Photocatalytic Dye Degradation

Kamal Prasad Sapkota [1,2], Md. Akherul Islam [3], Md. Abu Hanif [3], Jeasmin Akter [1], Insup Lee [1] and Jae Ryang Hahn [1,4,*]

1 Department of Chemistry, Research Institute of Physics and Chemistry, Jeonbuk National University, Jeonju 54896, Korea; mychemistry2037@gmail.com (K.P.S.); tina44445@gmail.com (J.A.); jrhstn@gmail.com (I.L.)
2 Department of Chemistry, Amrit Campus, Tribhuvan University, Kathmandu 44618, Nepal
3 Department of Bioactive Material Sciences, Jeonbuk National University, Jeonju 54896, Korea; akherulraju@gmail.com (M.A.I.); hanif4572@gmail.com (M.A.H.)
4 Textile Engineering, Chemistry and Science, North Carolina State University, 2401 Research Dr., Raleigh, NC 27695, USA
* Correspondence: jrhahn@jbnu.ac.kr

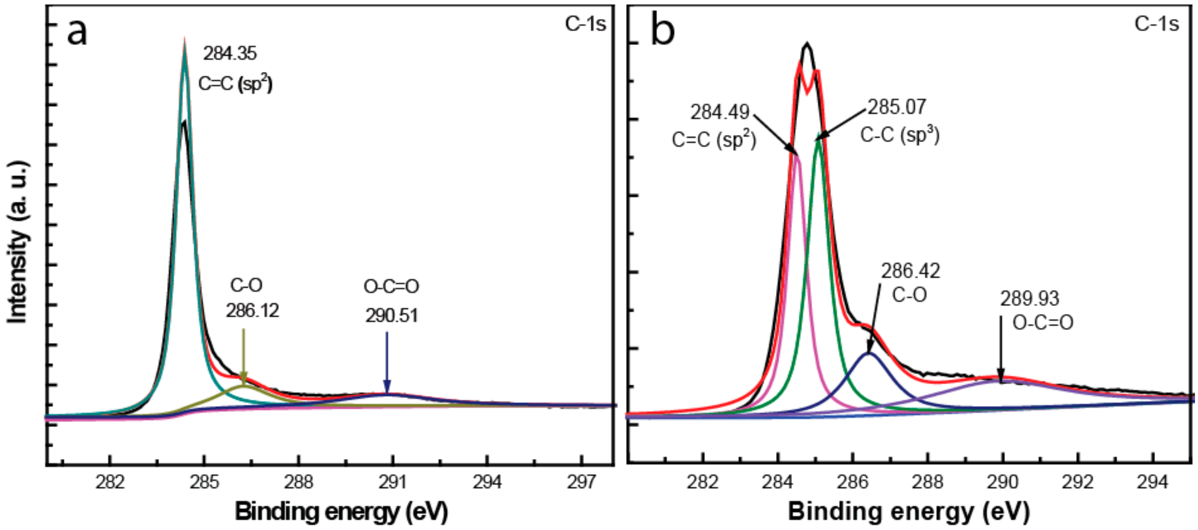

Figure S1. C-1s core level spectra of: (**a**) CuOSC-1 and (**b**) CuOSC-2 nanocomposites.

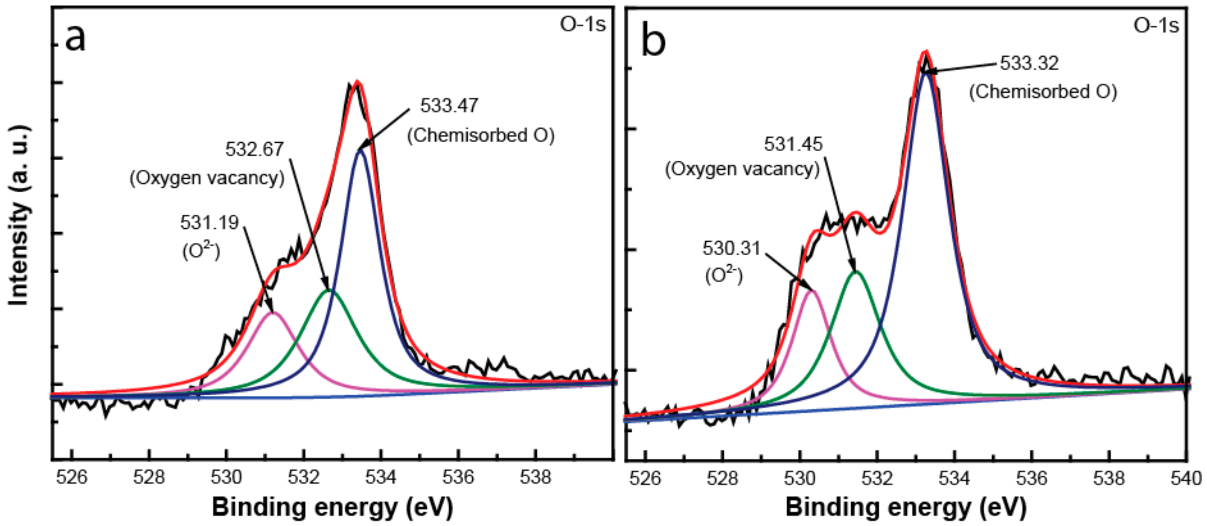

Figure S2. O-1s core level spectra of: (**a**) CuOSC-1 and (**b**) CuOSC-2 nanocomposites.

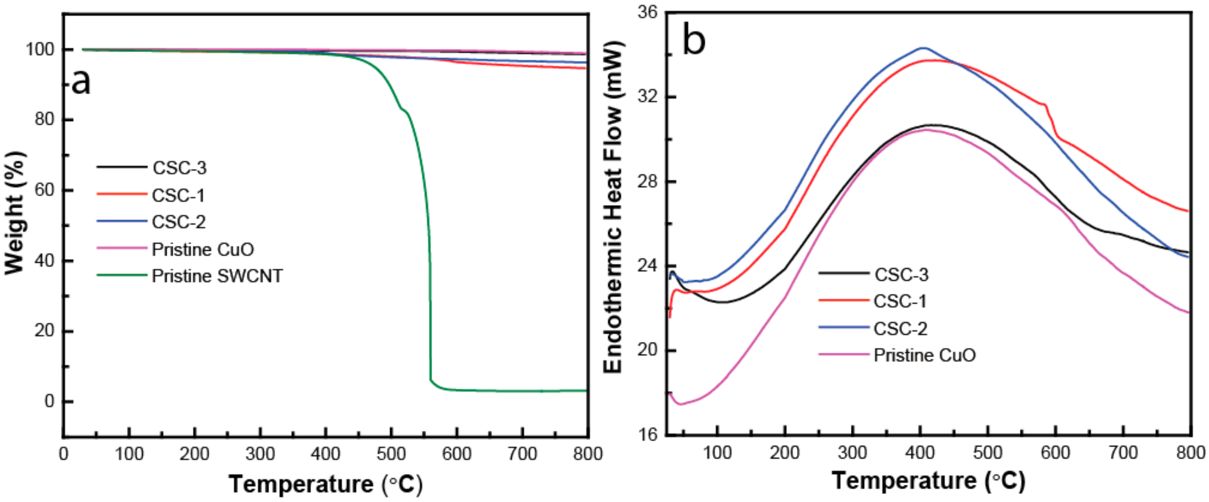

Figure S3. Analysis of the thermal stability of pure CuO, pure SWCNTs, and their nanocomposites: (**a**) TGA curves and (**b**) DSC thermograms.

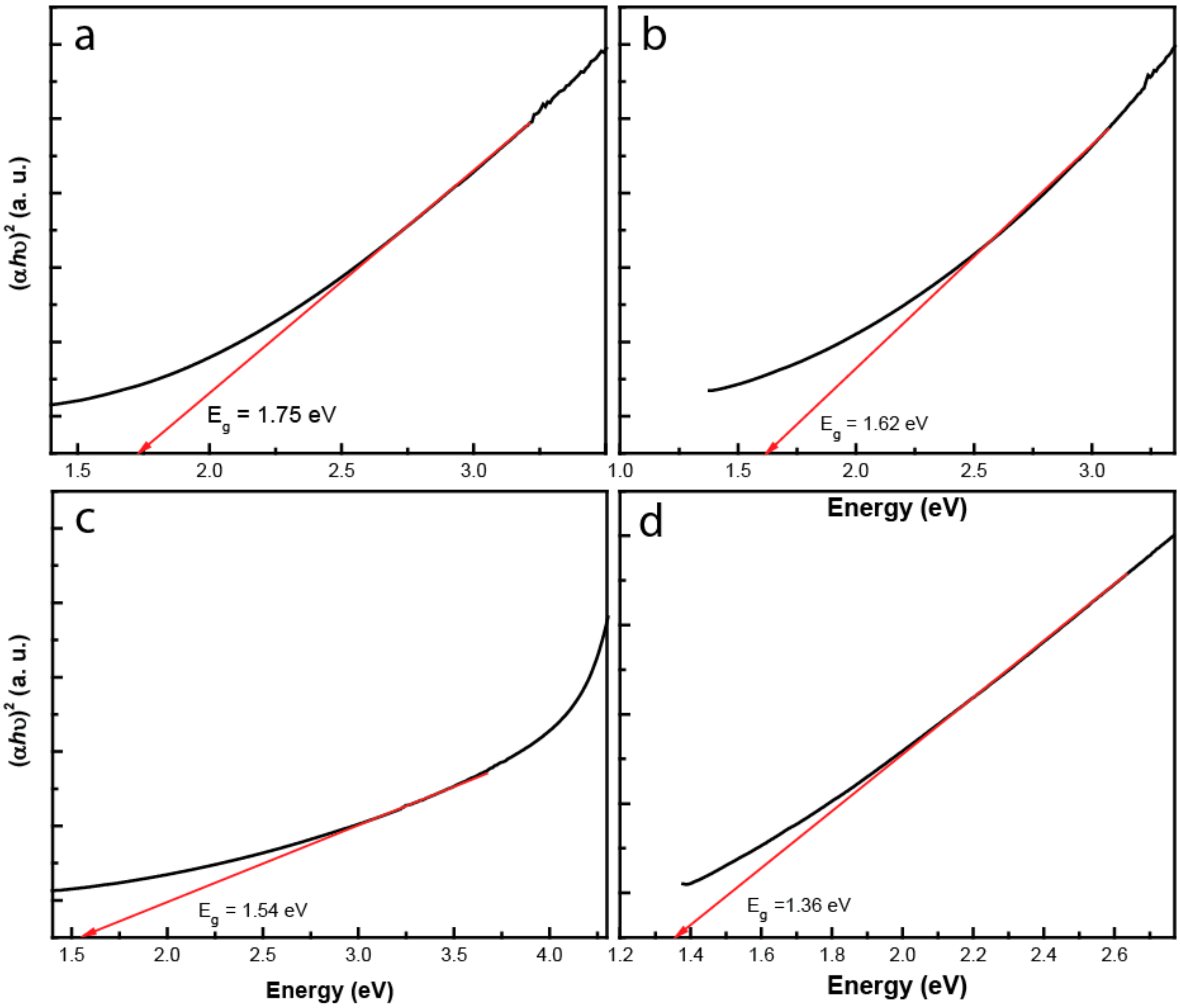

Figure S4. Tauc plots show a reduction of the bandgap of CuO owing to the creation of heterojunctions with SWCNTs: (**a**) Pristine CuO, (**b**) CuOSC-1, (**c**) CuOSC-2, and (**d**) CuOSC-3.

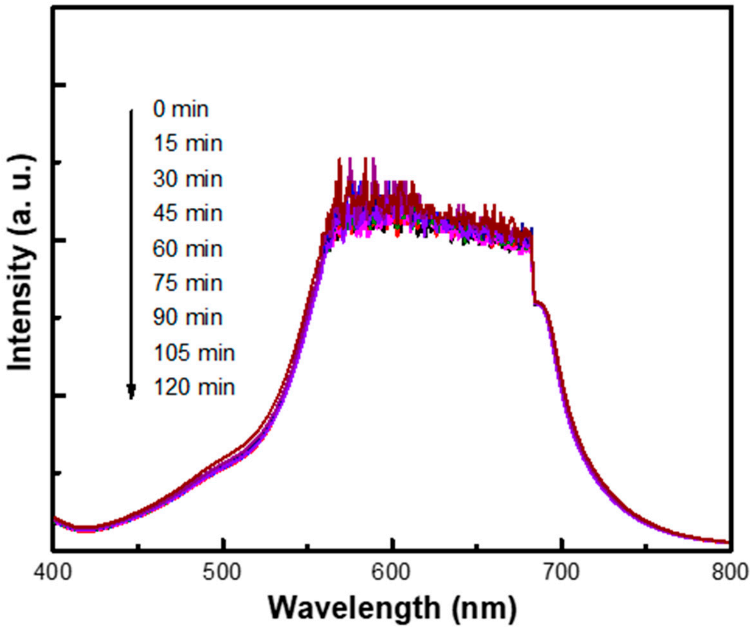

Figure S5. Blank test; effect of solar irradiation alone for the decomposition of MB in the absence of any catalyst.

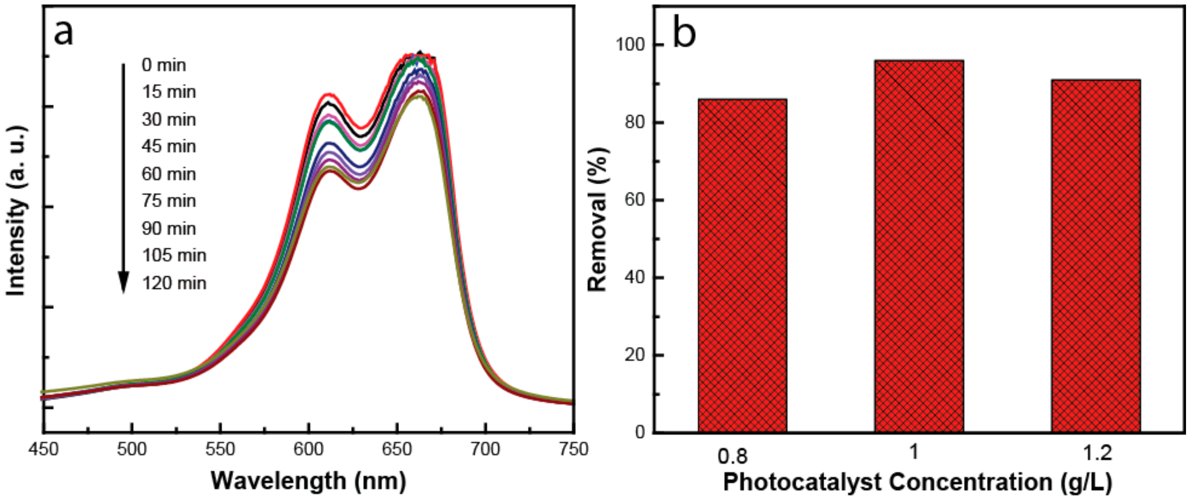

Figure S6. (**a**) Catalytic action of pure SWCNTs, and (**b**) Optimization of effective concentration of the CuOSC-3 photocatalyst displaying 1 gL^{-1} dose as the optimum concentration for the best photocatalytic action.

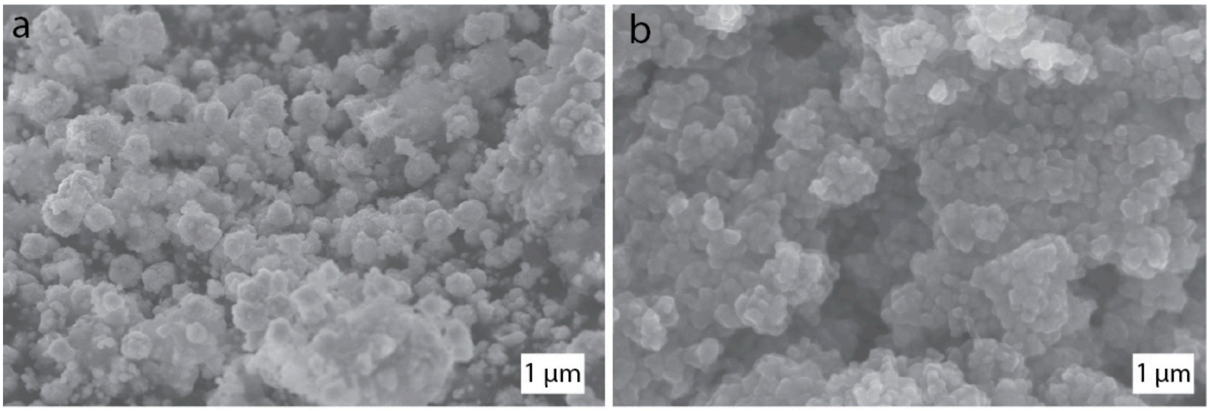

Figure S7. FE-SEM images of CuOSC-3 after fifth recycle, revealing the minor changes in the morphology of the nanocomposite.

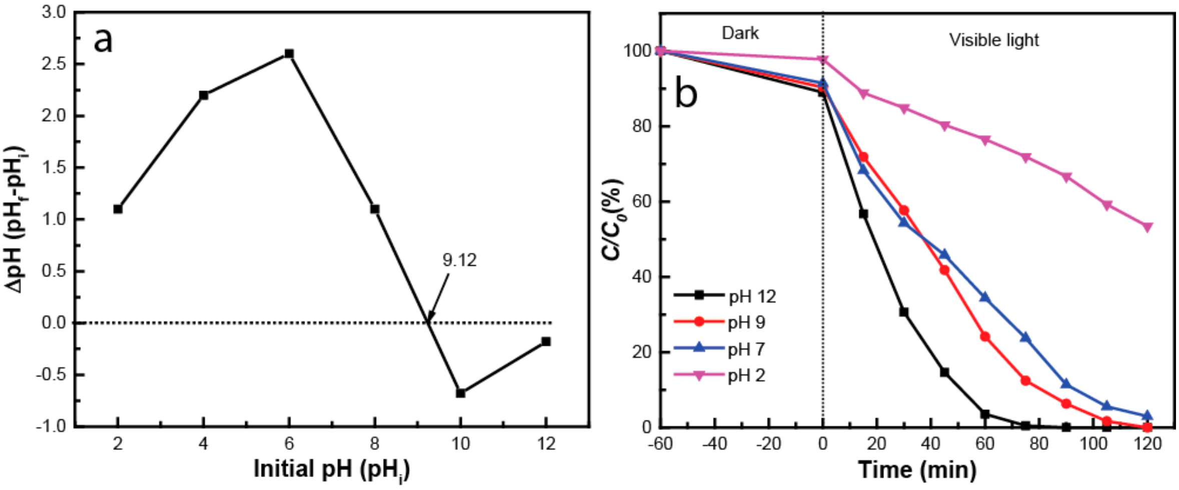

Figure S8. (**a**) Determination of the point of zero charge of the CuOSC-3 photocatalyst, and (**b**) effect of pH on the photodecomposition of MB by CuOSC-3.

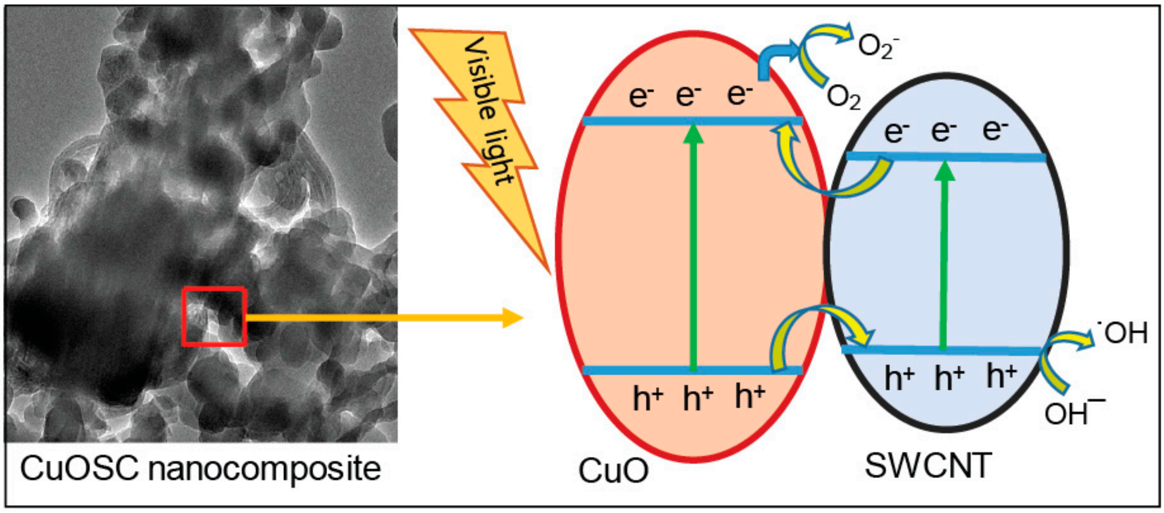

Figure S9. Proposed mechanism of generation of reactive oxygen species due to the irradiation of CuOSC nanocomposites with visible light.

Mechanism of photocatalytic action:

When the heterojunction nanocomposite is irradiated with visible light, each component of the composite absorbs energy whereby their electrons in the valence band (VB) are excited and jump to the conduction band (CB).

$$SWCNT + hv \rightarrow SWCNT_{(CB)}\ e^{-} + SWCNT_{(VB)}\ h^{+} \quad (1)$$

$$CuO + hv \rightarrow CuO_{(CB)}\ e^{-} + CuO_{(VB)}\ h^{+} \quad (2)$$

Photogenerated electrons from SWCNT (CB) instantly migrate to CuO (CB), whereas the holes from CuO (VB) move to SWCNT (VB) via the heterojunction formed between them. Thus, the photogenerated electrons and holes are separated and prohibited from recombination. The excess of free electrons attack dissolved O_2 and turn it into superoxide radical (Equation (3)).

$$e^{-}_{(CB)} + O_2 \rightarrow {}^{\cdot}O_2^{-} \quad (3)$$

The holes (h^+) react with surface-adsorbed hydroxyl groups (OH^-) from water to generate OH radicals, which attack MB molecules (Equation (4)).

$$h^{+} + OH^{-} \rightarrow {}^{\cdot}OH \quad (4)$$

The h^+ also attack water molecules to generate hydroxyl radicals and hydrogen ions (H^+). Then the combined actions of e^- and h^+ initiate a number of other reactions (Equations (5)–(10)) which are responsible for the decomposition of MB.

$$h^{+}_{(VB)} + H_2O \rightarrow H^{+} + {}^{\cdot}OH \quad (5)$$

$${}^{\cdot}O_2^{-} + H^{+} \rightarrow {}^{\cdot}HO_2 \quad (6)$$

$${}^{\cdot}HO_2 + H^{+} + e^{-} \rightarrow H_2O_2 \quad (7)$$

$$H_2O_2 + e^{-} \rightarrow {}^{\cdot}OH + OH^{-} \quad (8)$$

$$H_2O_2 + hv \rightarrow 2\ {}^{\cdot}OH \quad (9)$$

$${}^{\cdot}OH + MB \rightarrow {}^{\cdot}R\ (\text{Intermediates}) \rightarrow CO_2 + H_2O \quad (10)$$

The h^+ can invade the MB molecules directly to split them via oxidation (Equation (11)).

$$h^{+} + MB \rightarrow {}^{\cdot}R\ (\text{Intermediates}) \rightarrow CO_2 + H_2O \quad (11)$$

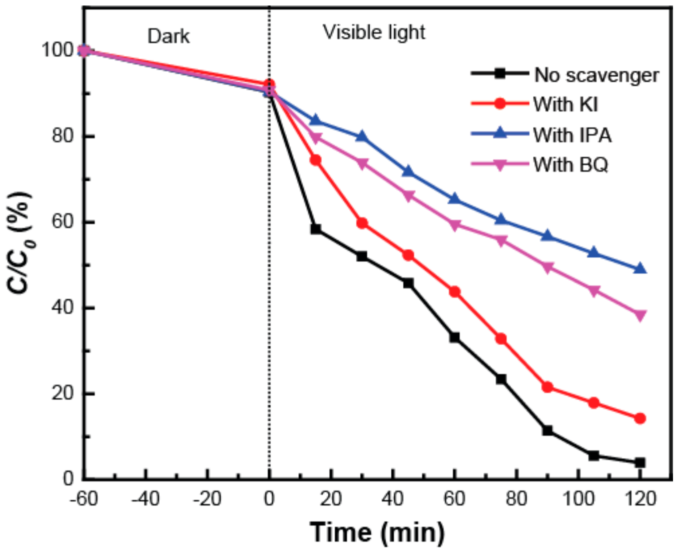

Figure S10. Effect of charge carrier scavengers on the photocatalytic decomposition of MB.

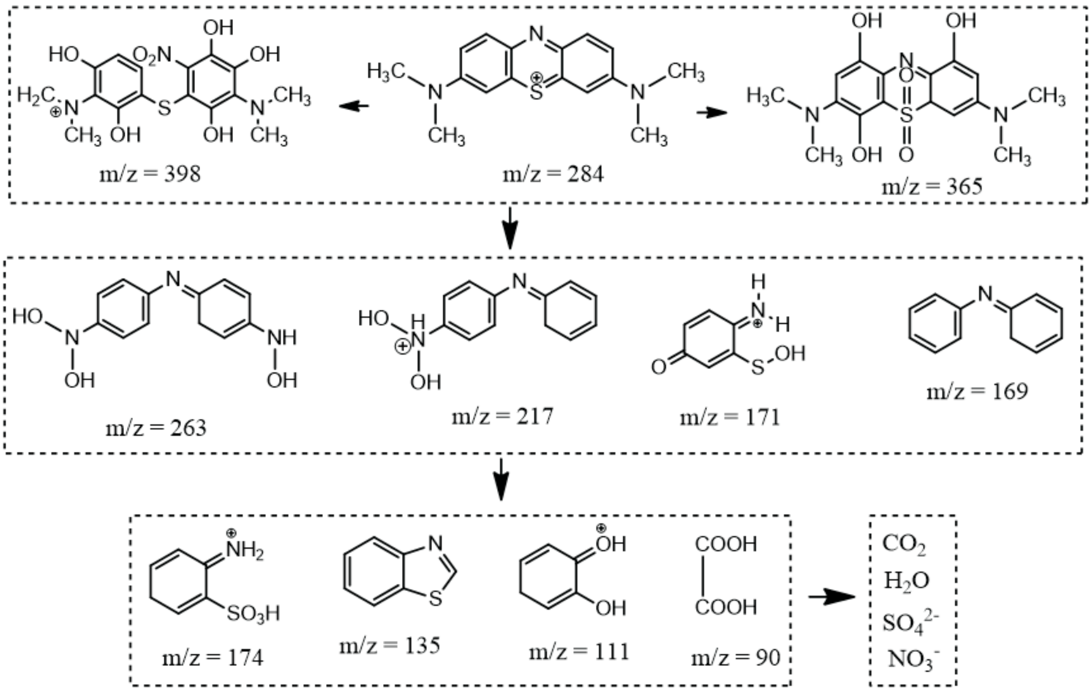

Figure S11. Proposed mechanism of degradation of MB into intermediates leading to the formation of harmless products because of the photocatalytic action of CuOSC nanocomposites under visible light irradiation.
